# Risk Factors for Children's Vocabulary Performance: Background Variables and Parental Concerns

**DOI:** 10.1111/1460-6984.70211

**Published:** 2026-02-18

**Authors:** Lina Hashoul‐Essa, Sharon Armon‐Lotem

**Affiliations:** ^1^ The English Literature and Linguistics Department Bar‐Ilan University Ramat‐Gan Israel; ^2^ The Multidisciplinary Brain Research Center Bar‐Ilan University Ramat‐Gan Israel

**Keywords:** language acquisition, palestinian arabic, parental concerns

## Abstract

**Purpose:**

This study aims to examine differences in vocabulary performance among Arabic‐speaking children ages 1;6–3;6, using a three‐domain theoretical framework (biological/genetic, developmental, and social/environmental factors), with particular focus on how these factors differ based on the presence of parental concerns.

**Method:**

Data were collected from 874 parents of Palestinian Arabic‐speaking children aged 18–36 months using the online Palestinian Arabic Communicative Development Inventory (PA‐CDI). An accompanying background questionnaire was used to gather information on five potential risk factors.

**Results:**

Children with parental concerns demonstrated significantly lower vocabulary performance across all age groups. The proportion of concerned parents decreased as vocabulary percentile increased. All five examined risk factors correlated significantly with vocabulary performance. Parental concerns emerged as the strongest predictor overall. For children without parental concerns, onset of speaking was the only significant predictor, while for children with concerns, word combination abilities were the sole significant predictor.

**Conclusions:**

Our findings highlight the validity of parental concerns as indicators of potential language difficulties and underscore the importance of early language milestones in predicting vocabulary performance. Word combination abilities, in particular, seem valuable for identifying children with persistent language difficulties. The PA‐CDI, combined with background variables, shows promise as an effective clinical tool for early identification of children at risk for language delays in Arabic‐speaking populations.

**WHAT THIS PAPER ADDS:**

*What is already known on this subject*

- Research has consistently demonstrated the validity and importance of parental concerns in identifying children's developmental issues, particularly in the early detection of DLD. Previous studies have established that parental observations can be as reliable as quality developmental screening tests. Early language milestones, including onset of speaking and word combinations, have been identified as significant predictors of vocabulary development. Risk factors for language development have been categorised into three domains: biological/genetic factors (family history, health conditions), developmental indicators (delayed milestones), and social/environmental factors. The CDI has emerged as a valuable tool for assessing vocabulary development and identifying children at risk for DLD across various languages.

*What this study adds to existing knowledge*

- This study reveals that parental concerns emerge as the strongest predictor of vocabulary performance in Arabic, an understudied language in this context, accounting for 27.2% of variance in language development. For the first time, we demonstrate differential predictive patterns between children with and without parental concerns: Onset of speaking predicts performance for children without concerns, while word combination abilities serve as the sole significant predictor for children with concerns. The study validates a three‐domain framework in Arabic‐speaking populations and shows that subjective factors demonstrate significantly stronger relationships with vocabulary performance than objective indicators, challenging traditional assumptions about measurement reliability.

*What are the potential or actual clinical implications of this work?*

- These findings highlight the value of considering parental perspectives alongside standardised assessments to gain a comprehensive understanding of children's linguistic abilities in Arabic‐speaking populations. Healthcare professionals should recognise that parental reports, when systematically collected, may provide more sensitive indicators of language development than traditional objective measures. Clinical assessment protocols should be restructured to give greater weight to structured parental observations. Word combination abilities, in particular, seem valuable for identifying children with persistent language difficulties. The PA‐CDI, combined with background variables, shows promise as an effective clinical tool for early identification of children at risk for language delays in Arabic‐speaking populations.

## 1 Introduction

Language development forms the foundation for communication, learning, and social interaction in children's lives. During the process of language acquisition, children undergo a series of distinct developmental stages. The rate at which they achieve each milestone varies between individuals, with some children reaching these stages faster than others (Bates et al. 1994). Nevertheless, there is usually a predictable sequence that children follow in their language development journey.

In examining factors that influence language development, research has identified three main domains of risk factors: (1) biological/genetic factors, including family history of language difficulties and health conditions affecting hearing or neurodevelopment; (2) developmental indicators, including delayed achievement of early language milestones such as first words and word combinations; and (3) social/environmental factors, including socioeconomic variables and parental awareness of development (Bates et al. 1994; Norbury et al. 2016). With this in mind, we shall use a three‐domain framework to provide structure for understanding the multifaceted nature of language development and potential delays, allowing for more targeted approaches to early identification and intervention. Within the three‐domain framework, risk factors can be further categorised as either subjective or objective measures. Subjective factors rely on parental report and perception, including onset of speaking, word combination abilities, and the parental concerns themselves. In contrast, objective factors include more concrete factors, such as documented family academic difficulties and medical health issues.

Developmental Language Disorder (DLD), affecting approximately 7% of children, is characterised by atypical language development that cannot be accounted for by cognitive, social, emotional, or motor abilities (Norbury et al. 2016). Children with DLD often experience delays in first words and word combinations compared to their typically developing peers, which is not secondary to any apparent underlying disorder (Trauner et al. 2000). In this study, we differentiate between risk factors, which could be biological/genetic, developmental, or social/environmental factors found to be specific indicators that increase the likelihood of DLD, and background variables (broader demographic and other environmental characteristics).

### 1.1 Background Variables as Risk Factors for Language Development

Our three‐domain framework provides a systematic approach to understanding how different types of risk factors contribute to language development. Each domain represents distinct mechanisms through which language difficulties may emerge.

Within the biological/genetic domain, hearing difficulties and repeated ear infections have been identified as factors contributing to delays in language development. Numerous studies have demonstrated that recurring ear infections impact vocabulary growth, grammar usage, sentence structure, and storytelling abilities (Feagans et al. 1987). Badran et al. (2013) observed that children with persistent middle ear infections exhibited lower scores in receptive, expressive, and overall language age. Similarly, Shriberg et al. (2000) discovered a connection between hearing levels at 12–18 months and both speech delays and diminished language proficiency at age three. However, other researchers have found no contribution of this factor (Dale et al. 2003; Jago et al. 2023).

Family history, reflecting genetic predisposition, has also been identified as a factor predicting language development and delay. among children Research has proposed a genetic link to DLD. In an investigation involving the KE family, Gopnik and Crago (1991) reported the discovery of a dominant gene, persisting through three generations, which affected half of the study's participants; specifically, 16 out of 30 family members exhibited dysphasia (what is now commonly known as DLD). Studies have highlighted that children with a familial background of DLD achieved lower scores compared to those without any history of DLD (Tallal et al. 1989; Spitz et al. 1997; Rice et al. 1998; Choudhury and Benasich 2003; Rudolph and Leonard 2016). Reilly et al. (2010) studied twelve biological and environmental factors and found that family language problems, along with two other factors, were successful in discriminating between children with and without expressive DLD at the age of four. Additionally, twin‐based studies have established that genes play a more substantial role than the environment in language disorders (Bishop et al. 1995; Rice et al. 2014, 2018). Hashoul‐Essa (2017) found that children who appeared to be at risk for DLD had a sibling with DLD. On the other hand, other recent studies have found no effect of familial language difficulties (Auza et al. 2023a; Jago et al. 2023).

In the developmental domain, the age at which children begin producing first words and progressing to two‐word combinations is considered a significant milestone. The one‐word stage typically begins around the end of the first year (12–18 months), followed by first word combinations between 18 and 24 months. Subsequently, around 24 to 30 months, children progress to producing simple sentences, and later, at 30 months and beyond, they begin to construct more complex sentences (Clark 2009). Children with DLD show delays in these milestones compared to their typically developing peers (Trauner et al. 2000). Rescorla (1989) found that for children who were 2 years old, fewer than 50 vocabulary words or no word combinations was the most accurate indicator for identifying language delays. Rudolph and Leonard (2016) found that delayed acquisition of word combinations (after 24 months) was a better predictor of DLD compared to delayed acquisition of first words (after 15 months). Likewise, Auza et al. (2023a) also found no contribution from age of onset of first words, while Sansavini et al. (2021) found a contribution for word combining.

### 1.2 Parental Concerns of Language Development

In the social/environmental domain, parental concerns represent a key factor in early identification. Parents naturally compare their children's progress to that of their social network, which can raise concerns if they perceive their child as falling behind. They may notice that their child does not meet typical language milestones and struggles with language, showing difficulty understanding instructions or expressing themselves clearly. Concerns about such delays in expressive language are often expressed by parents when seeking professional help (Rescorla and Lee 2000; Rescorla and Alley 2001; Rescorla 2011). These delays do not necessarily indicate a problem. Late talkers may eventually catch up over time. However, some late‐talking toddlers continue to have language delays, and 40%–50% are later diagnosed with DLD (Bishop 2017; Rescorla 2011).

Research has consistently demonstrated the validity and importance of parental concerns in identifying children's developmental issues, particularly in early detection of DLD. Glascoe et al. (1989) observed a strong correlation between parental concerns and children's performance on developmental screening tests. Their study revealed that 88% of children whose parents expressed concerns failed these tests, while 94% of children without parental concerns passed, highlighting the predictive power of parental observations. This finding was further supported by Glascoe (1999a), who found that parents from diverse backgrounds, regardless of education, socioeconomic status, or parenting experience, could accurately pinpoint their children's developmental challenges. The accuracy of parental concerns has been shown to rival that of professional assessments. Glascoe (1999b) found parental concerns to be as reliable as quality developmental screening tests, while Harris (1994) demonstrated that parents' responses to just four questions about their children's overall development were nearly as accurate as professional evaluations. Recent studies have further reinforced the value of parental input in identifying language disorders. Auza et al. 2023a, 2023b) developed a parental language concern questionnaire that proved effective as a screening tool for DLD. Similarly, Peñaloza et al. (2021) found that parental reports provided crucial support in the early identification of children with persistent language difficulties.

### 1.3 Using the CDI in Assessment of Risk for DLD

The MacArthur–Bates Communicative Development Inventories (MB‐CDI), introduced by Fenson et al. (1994) and adapted into more than 60 languages, tap into parents' knowledge of their children's early lexical and morpho‐syntactic language development. Research has demonstrated the reliability of the MB‐CDI in assessing children's language development (Fenson et al. 1994, Fenson et al. 2000). This reliability extends to adaptations to Semitic languages (Abdelwahab et al. 2021; Gendler‐Shalev and Dromi 2022; Hashoul‐Essa and Armon‐Lotem 2024). It has emerged as a valuable tool not only for assessing vocabulary development but also for identifying children at risk for DLD. Thal et al. (1999) confirmed the CDI's validity for assessing vocabulary production in language‐delayed children aged 24 to 32 months, revealing strong correlations between parent‐reported vocabulary and behavioural measures. This validity extended to older children (39–49 months) for both vocabulary and grammar production. Dale et al. (2003) established a criterion for identifying late talkers at risk for DLD, using the 10th percentile cut‐off on CDI vocabulary production scores at age 2.

Subsequent studies have further refined and validated the CDI's diagnostic capabilities. Heilmann et al. (2005) found significant correlations between CDI scores and direct language measures in 30‐month‐olds, effectively identifying children with low language skills (below the 11th percentile) and those with normal skills (above the 49th percentile). They proposed a 19th percentile cut‐off for screening purposes, enhancing the CDI's practical application. The CDI's effectiveness has been demonstrated across different languages and contexts. Sachse and Von Suchodoletz (2008) used the CDI alongside direct assessments to evaluate language abilities in German‐speaking toddlers, confirming its utility for early identification of language disorders. In the Arabic‐speaking context, Hashoul‐Essa (2017) successfully employed the PA‐CDI to identify children below the 10th percentile who appeared at risk for DLD. Recent research by Eriksson and Myrberg (2023) on the Swedish CDI further corroborated these findings, showing that children diagnosed with language disorders scored below the 10th percentile on expressive vocabulary scales.

Parental concerns and report questionnaires play a crucial role in the early identification of language delays in children (Glascoe and Dworkin 1995; Chung et al. 2011). Recent research has further emphasised their predictive power and importance in assessing language development risks. Tuller (2015) supports this approach, particularly in multilingual contexts, arguing that these tools effectively gather essential information about a child's language use across different environments. For example, Hashoul‐Essa and Armon‐Lotem (2024) found that parental concerns and health issues significantly predict both lexical and morphosyntactic development, with an inverse relationship between concern levels and language scores. For bilingual children, Abutbul‐Oz and Armon‐Lotem (2022) stress the importance of parental input in the diagnostic process, suggesting that parent questionnaires provide valuable insights into developmental milestones and language exposure. By integrating parental observations, clinicians can achieve a more comprehensive understanding of a child's language profile, leading to more accurate diagnoses and effective interventions. Additionally, health issues such as ear infections were found to negatively impact language development, likely due to their effect on a child's ability to process spoken language.

### 1.4 The Current Study

The current study utilizes the web‐based Palestinian Arabic Communicative Development Inventory (PA‐CDI) (Hashoul‐Essa and Armon‐Lotem 2024), developed for children aged 18–36 months and adapted for various Palestinian Arabic dialects in Israel. This tool, along with an accompanying background information questionnaire, allows a comprehensive assessment of children's vocabulary performance and early developmental milestones.

In addition to parental concerns, previous studies explored biological/genetic, developmental, and social/environmental risk factors to determine whether considering such factors may lead to a better identification of DLD and improve the precision of the CDI. Yet, results vary and are sometimes inconclusive as to the contribution of different factors to the diagnosis of DLD. The current study focuses on what parents report regarding their children's language development, exploring the impact of 5 of the potential risk factors: three subjective factors (onset of speaking, onset of word combination, and parental concerns) and two objective factors (family academic difficulties and health issues) on language development, and discusses the use of parental reports as a valuable tool. Two main research questions are addressed:

1. Do children who have parental concerns indeed demonstrate lower vocabulary performance compared to children with no concerns?
2. Using our three‐domain framework of risk factors (biological/genetic, developmental, and social/environmental), which specific factors best predict the vocabulary performance of children? Are different factors relevant for children with and without parental concerns?

It is expected that the vocabulary performance of children who have parental concerns will be significantly lower than children whose parents do not have concerns. Moreover, it is expected that the proportion of children with and without concerns will be different for different percentiles of vocabulary performance, so that the higher the percentile is, the lower the proportion of children with parental concerns is. As for risk factors, it is expected that the five risk factors will have an effect on the vocabulary performance of children. It is also expected that the sum of these five factors will have the highest effect. Yet, for children with parental concerns, it is expected that family academic difficulties and health issues will predict vocabulary performance (Gopnik and Crago 1991; Badran et al. 2013), as late onset of speaking and word combination are likely to yield these concerns and thus correlate with it. By contrast, it is expected that for children without parental concerns, the onset of speaking and word combination will predict vocabulary performance.

## 2 Method

### 2.1 Participants

At the onset of the study, 3500 mothers of Palestinian Arabic‐speaking incomplete children residing in Israel were recruited through social media platforms. After excluding responses and those outside the target age range of 18–36 months, 1399 valid questionnaires remained. Of these, 874 respondents addressed the crucial question about concerns regarding their children's language development. This final sample comprised 570 mothers who reported no concerns and 304 who expressed concerns about their children's linguistic progress. All participating families indicated Arabic as their primary household language, with children having only limited exposure to English or Hebrew, mainly through cartoon viewing. Table 1 shows the participants’ distribution by age in months and parental concerns.

**TABLE 1:** Participants.

Age in months	Number of participants (875)	Percentage of concerned parents
18	65	17
19	64	28
20	42	26
21	58	38
22	39	41
23	39	49
24	48	35
25	46	31
26	49	55
27	52	44
28	53	38
29	42	38
30	47	30
31	55	42
32	40	35
33	34	29
34	34	29
35	30	30
36	38	26

### 2.2 Methodology

The PA‐CDI comprises two main sections: lexical and early non‐verbal cognitive development, and morphosyntactic and syntactic development. This study focused solely on the lexical component, which assesses the production of 658 vocabulary items across 21 categories. These categories encompass a wide range of concepts, from basic sounds and animals to more complex linguistic elements like pronouns and connectors. For more information on the second part on morphosyntactic development, see Hashoul‐Essa and Armon‐Lotem (2024).

Accompanying the PA‐CDI was a comprehensive background questionnaire designed to gather both demographic and developmental data. The demographic section collected information on the child's gender, birth order, and number of siblings, as well as parental education level (primary, secondary, university, or other professional training) and profession, as general background variables.

The questionnaire also addressed specifically biological genetic factors, asking explicitly about family history of academic difficulties with the question: “Has anyone in your immediate or extended family (child's siblings, parents, or relatives) experienced difficulties in school, reading, writing, understanding others, or expressing themselves?” Parents were asked to specify which family members experienced these difficulties and whether they had received speech‐language therapy. Likewise, health‐related questions addressed complications during pregnancy or birth, birth weight, and whether the child experienced any specific medical conditions. Parents were also explicitly asked, “Has your child experienced ear infections or hearing problems?” and were asked to indicate the frequency of ear infections (once, twice, three times, four times, five times, or more) and whether the child had received any special ear treatments. Additional health questions covered allergies and other conditions that might affect language development.

The developmental portion of the questionnaire focused on early linguistic and developmental milestones, specifically asking, “At what age did your child begin to walk?”, and “At what age did your child begin to say their first words?”, and “At what age did your child begin to combine two words?” with response options spanning five age ranges (up to 12 months, 13–18 months, 19–24 months, 25–30 months, and 31–36 months).

Finally, the questionnaire specifically asked parents about concerns regarding their child's language development with the question, “Do you feel that your child is delayed in language development?”, requiring a yes/no response. If parents answered “yes”, they were asked to provide further details about their concerns.

### 2.3 Procedure

Participant recruitment was conducted through a month‐long targeted advertising campaign on Facebook and Instagram. The ads were tailored to reach Arabic‐speaking women aged 18–45 residing in predominantly Arabic‐speaking areas and mixed communities across Israel. The web‐based PA‐CDI was optimised for both mobile and desktop platforms to ensure broad accessibility. Upon accessing the study website (http://www.pacdi.online), participants were presented with a detailed explanation of the questionnaire and instructions for completion. They were then prompted to provide informed consent for participation. The study was reviewed by the Ethics Committee at the university and determined to be exempt from full ethical review due to minimal risk to participants. While participants provided email addresses to receive their child's results, these identifiers were separated from response data during analysis to protect privacy and were not kept once the certificate was sent. All participants provided explicit informed consent for their anonymised data to be used for research purposes before accessing the questionnaire. After submitting basic information (email, child's birth date and gender) and consenting to anonymous data use for research purposes, participants progressed through three main sections: lexical development, in which parents identified words their child could produce from provided lists; morphosyntactic development, in which parents selected options that best matched their child's speech patterns and provided examples of the child's longest utterances; and background information, in which parents furnished demographic and developmental data. Upon completion of all sections, participants could generate a summary of their child's vocabulary size, serving as both a reward and a valuable reference for the parent.

### 2.4 Analysis

A database was curated from the questionnaires and was analysed for lexical development. The questionnaire has an automatic scoring system that leaves no space for the scorer's judgment, making the scoring simple and reliable. For lexical development, we looked at the overall number of words within each lexical and grammatical category. The raw scores were used within each age bracket (in months) to determine the 10th, 25th, 50th, 75th, and 90th percentiles specifically for this sample of Palestinian Arabic‐speaking children. Since standardised norms are not yet available for this population, sample‐based percentiles were assigned to each of the children by ranking their vocabulary scores within each monthly age group and determining the percentage of children scoring at or below each participant's score. A chi‐square analysis was also conducted to examine the association between the number of concerned parents and the number of unconcerned ones across the different percentiles.

To address the contribution of the five risk factors, we used three subjective factors that rely on parental report and perception (onset of speaking, word combination abilities, and parental concerns) and two objective factors that represent more concrete, verifiable indicators (family academic difficulties and health issues). All were turned into categorical variables with a score of 1 or 0. For the onset of speaking and word combinations, our cut‐off points were determined by the age at which 90% of children in our sample had achieved these milestones. Since 90% of children in our data had produced first words by 12 months, those reported to begin speaking after 12 months were classified as late word producers (score = 0). Similarly, as 90% of children had started combining words by 22 months, those combining words after 22 months were classified as late word combiners (score = 0). A score of 0 was also assigned for the presence of parental concerns, reported hearing problems or ear infections, or family members with language difficulties. The sum of these five factors for each child could range from 0 (indicating risk on all factors) to 5 (indicating no risk on any factor).

Statistical analyses were conducted using IBM SPSS Statistics (IBM Corp., 2024) and R (R Core Team, 2025). Due to the non‐normal distribution of vocabulary scores, Spearman correlations were conducted for the five risk factors to examine the relationships between child percentile performance and background variables. These were followed by linear regression analyses, conducted to determine which risk factors contribute to lexical development in general, and then to examine the contribution of the background variables in predicting vocabulary performance in children without parental concerns and children with parental concerns.

### 2.5 Findings

Findings are presented for all children dividing them by parental concerns and addressing the different risk variables. Table 2 provides descriptive information for the five risk variables whose effect on language development was tested.

**TABLE 2:** Background variables.

	Number	Percentage of study
Onset of speaking—Age (months) of talking one word (*n* = 815)		
Up to 12	751	92.1
13–18	50	6.1
19–24	12	1.5
25–30	2	0.2
31–36	0	0
Word combination—Age (months) of combining two words (*n* = 775)		
Up to 12	376	48.5
13–18	271	35.0
19–22	52	6.7
23–30	74	9.5
31–36	2	0.3
Reporting of parental concerns regarding child's language (*n* = 875)	304	34.7
Reporting of family academic difficulties (*n* = 886)	289	32.6
Reporting on health concerns of child (*n* = 880)	198	22.5

#### 2.5.1 Vocabulary Performance of Children and Parental Concerns

In order to examine whether there is a difference in the vocabulary performance of children who have parental concerns and children who do not, a *t*‐test was conducted to examine the potential differences in vocabulary performance between children with parental concerns (*n* = 304) and children without parental concerns (*n* = 571), regardless of age group. The results revealed a significant difference in vocabulary scores between the two groups (−18.124 (873), *p* = 0.000), indicating that children with parental concerns exhibited lower vocabulary performance compared to their counterparts without parental concerns. This significant difference was observed across all age groups, except for at the ages of 18 and 35 months.

**FIGURE 1:**
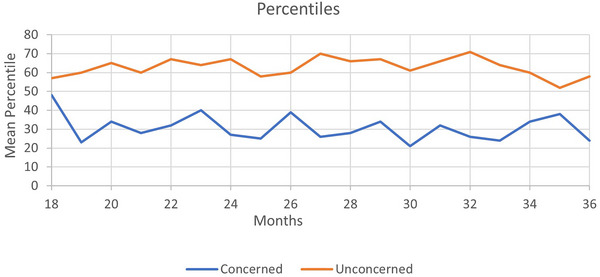
Mean percentiles of children with and without parental concerns.

Next, mean percentiles were calculated for each group based on the children's individual percentiles. Figure 1 shows the mean percentiles for each age group for children of concerned parents and children of unconcerned ones.

**C**hildren with parental concerns had lower average scores at each age group than children without parental concerns. The differences were significant across all age groups, except for at the ages of 18 and 35 months. Children with parental concerns had a mean percentile score of 30.6% (SD = 24%), whereas children without parental concerns had a higher mean percentile score of 63% (SD = 25.7%).

**FIGURE 2:**
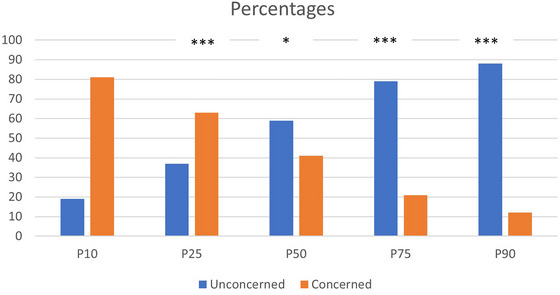
Percentages of concerned and unconcerned parents in each percentile.

In order to examine whether there is a difference in the proportion of children with and without concerns for the different percentiles of vocabulary performance, percentages of concerned and unconcerned parents across various percentiles were calculated for both groups. Figure 2 shows the percentage of concerned and unconcerned parents across various percentiles for the whole group.

Figure 2 shows that there are more concerned parents amongst the lowest percentiles and fewer concerned parents among the highest percentiles; that is, parents’ concerns get lower as children's vocabulary gets higher. A chi‐square analysis indicated statistically significant differences between concerned and unconcerned parents across all percentiles (*p* < 0.001 for the 90th, 75th, 25th, and 10th percentiles and *p* <0.05 for the 50^th^ percentile). This was true even for children who were in the 50^th^ percentile, where 41% of the parents were concerned.

2.5.2

In order to examine the relationships between child percentile and risk factors, Spearman correlation analysis was conducted for onset of speaking, word combination abilities, parental concerns, family academic difficulties, and health issues. The analysis was also conducted for the sum of these five factors. The results revealed significant correlations between children's percentile and all five examined risk factors, suggesting that children who had fewer risk factors exhibited higher vocabulary performance. An earlier onset of speaking was associated with higher vocabulary skills (*r* = 0.199**, *p* < 0.05), and the same was true for word combination (*r* = 0.243**, *p* < 0.01) and parental concerns (*r* = 0.519**, *p* < 0.05). Family academic difficulties demonstrated a very low yet significant positive correlation with child percentile performance (*r* = 0.075*, *p* < 0.05), and so did health issues (*r* = 0.081*, *p* < 0.05). Finally, a strong significant positive correlation was observed between child percentile performance and the sum of the five risk factors (*r* = 0.414**, *p* < 0.01).

In order to determine which risk factors contribute to lexical development, a general linear regression model was used, with the five risk factors as predictors and percentile as the dependent variable. VIF values for all regression models were below the acceptable threshold of 5 (below 2). Table 3 outlines the variables and their relative contribution.

**TABLE 3:** Background variables contributing to lexical development.

Predictor	*B*	*t*
(Intercept)	15.05	3.45^***^
Family academic difficulties	0.43	0.22
Health issues	4	1.80.
Onset of speaking	9.48	2.61^**^
Word combination	7.69	2.23^*^
Parental concern	28.41	13.42^***^
Model summary		
*R* ^2^	0.272	
*F* (5, 729)	54.39^***^	

*p < 0.05.

**p < 0.01.

***p < 0.001.

The model predicted 27.2% of the variance in vocabulary performance (*F* = 54.39 (729), *p* < 0.001). Parental concerns were the strongest predictor (*p* = 13.42***) of lexical development. Onset of speaking (*p* = 2.61**) and word combination abilities (*p* = 2.23*) also emerged as significant predictors of vocabulary performance. Academic difficulties of family members (p = 0.22) and health issues (*p* = 1.80) did not significantly contribute to the model.

Parental concern, word combination abilities, and onset of speaking are three measures that might be related to the dependent variable (vocabulary size) and are reported by the same parent. Thus, they are vulnerable to being subjective and biased. In order to further understand the contribution of the risk factors, subjective variables (onset of speaking, word combinations and parental concerns) and objective biological/genetic variables (family academic difficulties and health issues) were introduced into two separate regressions.

Table 4 outlines the separate contribution of subjective and objective variables:

**TABLE 4:** Separate regression analyses for subjective and objective predictors of vocabulary performance.

	Subjective	Objective		
Predictor	*B*	*t*	*B*	*t*
Onset of speaking	10.04	2.78**	—	—
Word combination	8.25	2.39*	—	—
Parental concern	28.98	13.86***	—	—
Age in months	0.13	0.74	—	—
Family academic difficulties	—	—	4.36	2.07*
Health issues	—	—	5.5	2.32*
Model summary		
*R* ^2^	0.274	0.012
*F*	69.30***	5.18*
*df*	4, 736	2, 873

*p < 0.05., **p < 0.01., ***p < 0.001.

For the subjective variables model (onset of speaking, word combination, and parental concerns), the analysis revealed that all three predictors significantly contributed to vocabulary performance. The model predicted 27.4% of the variance in vocabulary performance (*F* = 69.30 (4, 736), *p* < 0.001). Parental concerns emerged as the strongest predictor (*t* = 13.86***), followed by word combination abilities (*t* = 2.39**) and onset of speaking (*t* = 2.78***). Age in months was included as a control variable but did not reach significance (*t* = 0.74).

For the objective variables model (family academic difficulties and health issues), both predictors significantly contributed to vocabulary performance, though with considerably less explanatory power. The model predicted 1.2% of the variance in vocabulary performance (*F* = 5.18** (2, 873), *p* < 0.01), and family academic difficulties (*t* = 2.07**) and health issues (*t* = 2.32**) both emerged as significant predictors of vocabulary performance.

Due to the high contribution of parental concerns and the fear that parental concerns might overshadow the contribution of other variables, the two groups were next compared on the other risk factors. Table 5 outlines the difference between the two groups.

**TABLE 5:** Background variables of children of concerned & unconcerned parents.

	Concerned parents *N* = 304	Unconcerned parents *N* = 571	*t* / *χ* ^2^	Cohen's d
Average age (months) of onset of speaking	9.9 months	8 months	*t* = 7.993***	0.592
Average age (months)of word combining	17 months	12.8 months	*t* = 11.884***	0.913
Number of children who have members of family with academic difficulties	118 (39%)	171 (30%)	*χ* ^2^ = 9.720*	
Number of children who have health issues	83 (27%)	111 (19%)	*χ* ^2^ = 5.172**	

*p < 0.05., **p < 0.01., ***p < 0.001.

While the analysis revealed a significant difference between children of parents with and without concerns on all measures, we wanted to examine whether the same variables predict the vocabulary performance of the children in each group. Table 6 presents a comparative analysis of the contribution of the 4 subjective (developmental) and objective (biological/genetic) variables to vocabulary performance, with separate models for children of parents with no concerns (unconcerned) and children of parents with concerns (concerned).

**TABLE 6:** Background variables contributing to vocabulary performance: Comparison between children with and without parental concerns.

	Unconcerned		Concerned	
Predictor	*B*	*t*	*B*	*t*
(Intercept)	38.61	4.44***	19.69	3.59***
Family academic difficulties	1.68	0.66	−1.92	−0.61
Health issues	4.77	1.62	2.21	0.65
Onset of speaking	12.85	2.20*	7.18	1.6
Word combination	7.82	1.06	7.7	2.04*
				
Model summary				
	Concern = “1”		Concern = “0”	
*Df*	4, 489		4, 236	
*R* ^2^	0.024		0.038	
*F* (*df*)	2.95* (*p* = 0.02)		2.30 (*p* = 0.06)	
*N* (after NA removal)	494		241	

*p < 0.05., **p < 0.01., ***p < 0.001.

For children of parents with no concerns, the model indicated that the combination of these variables accounted for a small proportion of the variance in vocabulary performance (*R^2^* = 0.024, *F* = 2.95*, *p* = 0.02). The only variable which significantly predicted vocabulary performance was onset of speaking (*t* = 2.20*), while the other variables, including word combination abilities (*t* = 1.06), family academic difficulties (*t* = 0.66), and health issues (*t* = 1.62), did not make a significant contribution to the model.

For children of parents with concerns, the model accounted for a slightly larger proportion of the variance in vocabulary performance (*R^2^* = 0.038), though the overall model approached but did not reach statistical significance (*F* = 2.30, *p* = 0.06). The results indicated that only word combination abilities made a significant contribution to vocabulary performance (*t* = 2.04*), while the other variables, including onset of speaking (*t* = 1.6), family academic difficulties (*t* = −0.61), and health issues (*t* = 0.65), did not make a significant contribution to the model.

## 3 Discussion

This study examined vocabulary performance in relation to parental concerns and background variables using a three‐domain framework, targeting biological/genetic, developmental and socio/environmental risk factors. The results reveal clear patterns of the way different types of risk factors contribute to language development and provide validation for the organisation within the model.

## 4 Vocabulary Performance

Our results supported the hypothesis that children with parental concerns would exhibit significantly lower vocabulary performance than those without such concerns. This finding underscores the validity of parental worries as indicators of potential language difficulties, reflecting parents' attunement to their child's developmental progress. When parents are confident in their child's language progress, it likely indicates that the child is meeting or exceeding developmental expectations. When they perceive any deviation from expected language milestones, they may naturally become concerned. The absence of significant differences at 18 and 35 months presents an intriguing deviation. At 18 months, this may reflect high variability in early language development, making it challenging for parents to distinguish between typical and atypical progress. The lack of difference at 35 months could indicate late bloomers catching up. These exceptions highlight the need for further investigation into age‐specific factors influencing parental concerns and their relationship to actual language abilities, potentially informing more targeted screening approaches.

## 5 The Three‐Domain Framework in Practice

The three‐domain framework proved effective in organising risk factors and understanding their relative contributions to vocabulary development. Each domain showed distinct patterns of influence, validating theoretical organisation while revealing practical implications for assessment and intervention. We hypothesised that five factors (onset of speaking, word combination, parental concerns, family academic difficulties, and health issues) will have an effect on the vocabulary performance of children.

This hypothesis was confirmed based on the results of Spearman correlation analysis. The findings revealed significant correlations between children's percentile performance and all five examined background variables, suggesting that children with fewer risk factors tended to exhibit higher vocabulary performance. Parental concerns exhibited the highest correlation. An earlier onset of speaking and stronger word combination abilities exhibited positive correlations with higher vocabulary performance, emphasising the critical role that these early milestones play in shaping a child's vocabulary acquisition. Family academic difficulties and health issues showed relatively low correlations with vocabulary performance, implying that their contributions might be secondary to the other examined variables.

The Spearman correlation analysis revealed strong significant correlations between child percentile performance and the sum of all five examined background variables. Although the correlation with parental concerns was higher than the sum, it still was a strong one. This finding underscores the importance of considering a combination of factors rather than isolating individual variables when examining language development.

## 6 Social/Environmental Domain: The Power of Parental Concerns

Parental concerns, representing the social/environmental domain, show the strongest association with vocabulary performance. These results suggest a clear trend in the distribution of concerned and unconcerned parents across various vocabulary percentiles. Indeed, a higher percentage of concerned parents is observed among the lower percentiles of vocabulary performance. This pattern aligns with expectations, suggesting that parents of children who have lower vocabulary scores are more likely to be concerned about their linguistic development. Conversely, as children's vocabulary performance increases, the percentage of concerned parents steadily decreases. Children with stronger vocabulary skills are less likely to trigger concerns, potentially reflecting parents' confidence in their language growth. This trend is particularly evident comparing the lower to the higher percentiles. For instance, while a substantial 81% of children in the 10th percentile have concerned parents, this percentage decreases to 12% for children in the 90th percentile. This inverse relationship between parental concerns and vocabulary percentiles suggests that as children's language skills advance, parents tend to express fewer concerns about their language development.

These findings are in line with previous research (Harris 1994, Glascoe and Dworkin 1995, Glascoe et al. 1999a, 1989, 1999b, Auza et al. 2023a), indicating that parental worries are not mere emotional responses but rather indicators of potential underlying language difficulties. When parents express concerns about their child's language development, it serves as a signal that there may be challenges or delays in the child's linguistic abilities. This heightened awareness prompts parents to seek support and intervention when they perceive delays in their children's vocabulary development.

## 7 Developmental Domain: The Importance of Language Milestones

Early language milestones showed the expected association with vocabulary scores. Children with an earlier onset of speaking and earlier word combination abilities demonstrated higher vocabulary performance. This might reflect better aptitude for language acquisition, on the one hand, but could also suggest that parents of children who start speaking and combining words earlier might engage them more actively in language‐rich environments from an early age, as has been observed by Rescorla and Fechnay (1996). Such exposure provides them with more opportunities to absorb and learn words from their surroundings, and as a result, they accumulate a greater number of words in their vocabulary over time. The current study is unable, though, to tease the directionality of this parent‐child engagement apart.

## 8 Biological/Genetic Domain: Indirect but Important Influences

The biological/genetic domain showed the most surprising results. Although health issues of hearing and repeated ear infections (Feagans et al. 1987; Shriberg et al. 2000; Badran et al. 2013) and familial language difficulties (Tallal et al. 1989; Bishop et al. 1995; Spitz et al. 1997; Rice et al. 1998; Choudhury and Benasich 2003; Rice et al. 2014, 2018; Reilly et al. 2010; Rudolph and Leonard 2016) have been identified as factors contributing to delays in the development of language skills, these two factors did not significantly contribute to the model. Dale et al. (2003) found no contribution to health issues concerning ear infections, Auza et al. (2023a) found no contribution to familial language difficulties, and Jago et al. (2023) found no contribution to either factor. One possible reason worth considering is the individual variability in how children respond to health issues or familial language difficulties. Some children may be more resilient and able to overcome these challenges, while others may not.

Yet, once the biological/genetic variables, which are also more objective, were separated from the subjective variables, their association with vocabulary size was revealed. This suggests that the strong predictive power of parental concerns can mask the more subtle contributions of biological/genetic factors when analysed together. While subjective measures capture current observable behaviours, biological factors may create underlying vulnerabilities that influence language learning capacity through different pathways. This finding indicates that both domains provide valuable but distinct information—subjective measures excel at identifying immediate concerns, while biological factors may be more informative for understanding long‐term risk and intervention planning.

Thus, a key finding of this study is the substantial difference in predictive power between subjective and objective risk factors. Subjective measures (parental reports of concerns, onset milestones) demonstrated significantly stronger relationships with vocabulary performance than objective indicators (documented family difficulties, health issues). This pattern suggests that parents' observational reports, despite potential subjectivity, capture developmentally relevant information that may not be fully reflected in more objective medical or family history indicators.

The superior performance of subjective measures challenges traditional assumptions about measurement reliability in clinical assessment. While objective measures are typically preferred for their presumed accuracy, our findings suggest that parental expertise gained through daily observation may provide more sensitive indicators of language development than formal documentation of risk factors. This has important implications for screening protocols, suggesting that structured parental reports should be weighed heavily in early identification processes.

## 9 Differential Risk Factor Patterns Between Children With and Without Parental Concerns

Examining the two groups separately, we found significant differences in the onset of speaking and in word combining for children of concerned parents and children of unconcerned ones. The effect size of the word combining ability demonstrates a larger effect than the onset of speaking, suggesting that the difference in word combining ability is more pronounced and has a greater practical significance between the two groups compared to the difference in the onset of speaking. Furthermore, the results of the chi‐square analysis demonstrate notable and statistically significant variations between parents who exhibit concerns and those who do not in relation to health problems and familial academic difficulties. A higher proportion of children whose parents express concerns have health problems and family members experiencing challenges related to academic performance when compared to children whose parents do not display such concerns, which further substantiates our previous findings on the valuable role of parental concerns.

We hypothesised that for children with parental concerns, family academic difficulties and health issues will predict vocabulary performance, whereas for children without parental concerns, onset of speaking and word combination will predict vocabulary performance. The linear regressions showed that for children without parental concerns, only the onset of speaking emerged as a significant predictor, whereas for children with parental concerns, word combination abilities emerged as the sole significant predictor.

The observation that the onset of speaking emerges as a stronger predictor of vocabulary performance for children without parental concerns suggests that the ability to begin speaking at an early age strongly contributes to higher vocabulary skills. Starting to speak at an earlier age allows children more time and exposure to engage in language interactions, which might also be richer, leading to increased vocabulary acquisition. Such a pattern has been observed for children with DLD, showing that parents may decrease communication with children who are less talkative or responsive (Vigil et al. 2005; Rescorla and Fechnay 1996). However, this relationship may be bidirectional, and the causal relation in typically developing children within our age range requires further investigation through longitudinal studies.

Moreover, our study underscores that delayed word combinations provide a more accurate predictor of DLD compared to the delayed onset of production. While both children with DLD and late talkers show a delay in the onset of language development (onset of first words), children with DLD also show a delay in word combinations, whereas late talkers catch up and follow other milestones like typically developing children (Weismer, S. 2017). Moreover, children of concerned parents who started combining words earlier also had larger vocabularies than those who started combining words later. Thus, our findings reaffirm the significance of word combining skills in identifying children who might be at risk for language disorders. It could further suggest that what led to parental concerns is not necessarily the onset of production but rather the onset of word combining. What our findings indicate is that the differentiating factor between late talkers and DLD might lie in the capability to form word combinations. This aligns with previous research that suggested that the absence or delay of word combining is often a distinguishing characteristic between children who are considered late talkers (Rescorla 1989) and those who might have DLD (Rudolph and Leonard 2016).

## 10 Limitations

Several limitations should be acknowledged. First and foremost, the distinction between biological/genetic and developmental factors overlapped with the objective/subjective distinction. Future studies might want to consider objective measures of development (e.g., healthcare providers' documentation) or subjective environmental measures (which are not biological/genetic), such as socioeconomic status and maternal education, which have been shown to influence both language development and parental reporting accuracy, to test for the subjective‐objective effects. The former information was out of the scope of the current study, while the latter was restricted by the use of an online tool that excluded parents without high school education. Future studies should incorporate these variables to provide a more comprehensive understanding of risk factor interactions. Furthermore, the cross‐sectional design limits our understanding of developmental trajectories. Longitudinal studies are needed to examine how these risk factors influence language development over time and to distinguish between transient delays and persistent difficulties.

Moreover, while our three‐domain framework provided a useful organisational structure for understanding risk factors, this study did not directly test interactions between domains. It is likely that biological factors influence how children respond to environmental input, or that environmental factors moderate the expression of genetic predispositions. Future research should examine these cross‐domain interactions more explicitly to develop a more complete understanding of how multiple risk factors combine to influence language development.

Another limitation is a possible tendency of concerned parents to report more conservatively than non‐concerned parents, potentially influencing our findings. However, the systematic correlation between parental concerns and vocabulary performance across percentiles—with concern rates decreasing from 81% in the 10th percentile to 12% in the 90th percentile—suggests parents are responding to genuine developmental differences rather than applying uniform reporting biases. The differential predictive patterns between children with and without parental concerns further indicate that these reports reflect distinct developmental profiles rather than general reporting tendencies. Notably, the use of a binary classification of parental concerns (yes/no) may oversimplify the complexity of parental worry. Future research should explore the multidimensional nature of parental concerns, including their severity, specificity, and underlying reasons.

Finally, the validity of our word combination timing data could be questioned, as 48.5% of children were reported to combine words by 12 months. This is considerably earlier than typically expected developmental milestones (Clark 2009), which may reflect possible misunderstanding of the question format by parents. Future studies should include examples or samples to help parents better understand what constitutes word combinations versus single words

## 11 Conclusions

The present study aimed to investigate the vocabulary performance of children in relation to parental concerns and background variables using a three‐domain framework. The study first established that children with parental concerns exhibit lower vocabulary performance compared to those without such concerns, reinforcing the notion that parental worries are indicative of potential language difficulties. The correlation between parental concerns and vocabulary performance, as well as the distribution of concerned parents across different vocabulary percentiles, demonstrates the connection between these concerns and a child's linguistic development. This relationship highlights parents' inherent sensitivity to their child's developmental milestones, prompting them to seek support when deviations are detected.

Our application of the three‐domain framework successfully organised diverse risk factors and revealed their relative contributions to vocabulary development. The framework demonstrated that social/environmental factors, particularly parental concerns, serve as the most powerful predictors of vocabulary performance, followed by developmental milestones, while biological/genetic factors showed more modest direct effects. This hierarchy suggests that while genetic background and health conditions may influence developmental potential, observable behaviours and parental awareness provide more immediate clinical value for identifying children at risk.

The framework also revealed that risk factors operate differently across children with varying developmental profiles. For children without parental concerns, developmental milestones (onset of speaking) were most predictive, while for children with concerns, word combination abilities became the critical factor. This differential pattern indicates that assessment approaches should be tailored based on initial clinical presentations rather than applying uniform screening protocols.

These findings support the continued use of this three‐domain framework in both research and clinical practice. Moreover, the striking difference between subjective (27.4% variance explained) and objective factors (1.2% variance explained) highlights the unique value of parental observation in language assessment. This finding suggests that clinical protocols should prioritise structured parental reports while continuing to document objective risk factors as supporting information.

## 12 Implications and Future Directions

Parental concerns play a crucial role in the early identification and intervention of linguistic difficulties in children. Healthcare professionals should consider parental concerns as important indicators when assessing a child's language development. The findings highlight the value of considering parental perspectives alongside standardised assessments to gain a comprehensive understanding of a child's linguistic abilities and allow for timely intervention. Early language milestones, particularly the onset of word combinations, should also be considered in combination with parental concern to further support the language delays. The differential predictive power of subjective versus objective factors has important clinical implications. Healthcare professionals should recognise that parental reports, when systematically collected, may provide more sensitive indicators of language development than traditional objective measures such as family history or medical records alone. This suggests that clinical assessment protocols should be restructured to give greater weight to structured parental observations while using objective factors as complementary information. Training programmes for clinicians should emphasise the interpretation and systematic collection of parental reports as a core clinical skill.

## Conflicts of Interest

The authors declare no conflicts of interest.

## Data Availability

The authors will provide the foundational data that support the findings of this article upon request.
